# Optimized spatial information for 1990, 2000, and 2010 U.S. census microdata

**DOI:** 10.1038/s41597-023-02859-9

**Published:** 2024-01-05

**Authors:** Christopher S. Fowler, James D. Gaboardi, Jonathan P. Schroeder, David C. Van Riper

**Affiliations:** 1 Department of Geography, Penn State University, State College, USA; 2 Geospatial Science and Human Security, Oak Ridge National Laboratory, Oak Ridge, USA; 3 IPUMS, University of Minnesota, Minneapolis, USA

**Keywords:** Geography, Sociology

## Abstract

We report on the successful completion of a project to upgrade the positional accuracy of every response to the 1990, 2000, and 2010 U.S. decennial censuses. The resulting data set, called Optimized Spatial Census Information Linked Across Time (OSCILAT), resides within the restricted-access data warehouse of the Federal Statistical Research Data Center (FSRDC) system where it is available for use with approval from the U.S. Census Bureau. OSCILAT greatly improves the accuracy and completeness of spatial information for older censuses conducted prior to major quality improvements undertaken by the Bureau. Our work enables more precise spatial and longitudinal analysis of census data and supports exact tabulations of census responses for arbitrary spatial units, including tabulating responses from 1990, 2000, and 2010 within 2020 block boundaries for precise measures of change over time for small geographic areas.

## Introduction

This paper reports on the results of a project to retroactively optimize the locations assigned to every person and housing unit surveyed in the 1990, 2000, and 2010 decennial censuses. Working within the FSRDC system, we leverage updated address locations from more recent versions of the Census’ Master Address File (MAF) as well as modern geocoding techniques and improved representations of census block boundaries to locate census respondents more precisely and consistently, thereby creating a framework for high-quality longitudinal spatial analysis of census data from 1990 through 2020. Our work makes relatively limited improvements to the 2010 data, which already benefited from the MAF/TIGER Accuracy Improvement Project (MTAIP) of 2002–2008, but it makes significant improvements to the earlier data. The existing spatial information for the 2000 census was highly imprecise, and the available information for the 1990 census included only census block identifiers and addresses with no geographic coordinates. While there remain limitations in the quality of the spatial data for some years and geographic areas, the new data product nevertheless provides optimized spatial information by drawing from the best available source for each individual census response.

The output data set, named Optimized Spatial Census Information Linked Across Time (OSCILAT), includes two types of spatial information for every person and housing unit record in the complete 1990, 2000, and 2010 census microdata: (1) optimized geographic coordinates (latitude and longitude) and (2) identifiers for the census blocks where those coordinates are located. Each of these types of information is “linked across time” in a different way. First, the optimized geographic coordinates are generally derived from the most recent corresponding source of census spatial information, e.g., by “linking” a 1990 microdata record to coordinates for the corresponding address in the 2020 version of the MAF. Second, OSCILAT “links” every microdata record to multiple census years’ geographic units by identifying not only a *contemporary* block ID (e.g., the 2000 block where a 2000 census respondent resided) but also 2010 and 2020 block IDs (e.g., the 2010 and 2020 blocks where a 2000 census respondent resided). Since 1990, every census reporting area has corresponded exactly to a set of blocks, so the OSCILAT block IDs can be used to associate 1990, 2000 or 2010 census responses directly with any higher level of 2010 or 2020 census geography (census tracts, counties, etc.), thereby facilitating longitudinal comparison with consistent spatial units. The spatial scope of potential applications is also broad given that the geographic coverage of OSCILAT is the entire United States for all three census years and includes Puerto Rico for 2000 and 2010.

This information opens many new opportunities for high-precision spatial analysis. OSCILAT is the first resource of any kind to directly support spatial analysis of the 1990 census at the resolution of individual residences, and OSCILAT’s improved spatial precision for 2010 and, especially, 2000 microdata will also enable more exact measurements of spatial relationships between census responses and other features. Uneven quality in spatial information across regions within the same year and across years in longitudinal analysis introduces bias that may be particularly pronounced when using small geographic units^1^. It is now possible to delineate precise spatial contexts and neighbor relationships for household- or individual-level analysis for any census back to 1990. It is also possible to generate high-quality aggregate data for custom units (e.g., zones designed to vary by specified demographic characteristics or levels of flood risk or pollution exposure) and for consistent units across time, supporting robust longitudinal analysis. Microdata work undertaken within the FSRDC system^2,3^ can now employ our OSCILAT data to improve the accuracy of information about highly localized migration patterns. Work using non-standard geographic boundaries (e.g., school attendance zones)^4^ can be brought into the FSRDC environment where counts can be established precisely for analysis.

Perhaps most significantly, as it affects the much larger research community working outside of the restricted-access FSRDC environment, OSCILAT will permit careful examinations of error in publicly available longitudinal geographic data sets, and the results of such assessments can benefit all users of these data sets. With every new census, there are widespread changes in the boundaries of census reporting areas, and where spatial units are not consistent across time, it can be impossible to measure exact population changes. To address this problem, several data providers have generated geographically standardized time series of census data^5–7^. The typical approach is to select one census year’s geographic units to be the standard units (e.g., 2010 census tracts) and then transform summary data from other years (e.g., 1990 and 2000 tract data) using some method of areal interpolation to estimate each year’s characteristics within the standard units (e.g., 1990 and 2000 populations of 2010 census tracts). Different providers use different methods and base data to generate their series, which can result in major differences in accuracy. People are spatially sorted at very small scales by, for example, race and income, but interpolation methods can obscure this sorting by using larger source units (e.g., tracts instead of blocks) and by assuming uniformity within each unit. Until now, with few exceptions, it has not been possible to assess the accuracy of longitudinal data products by comparison against ‘true’ counts^5,7^. OSCILAT enables something very close to this, supporting high-quality measurements of interpolation error for a wide range of characteristics across multiple years. We expect this will lead to improved public estimates and better guidance on their fitness for use.

## Background and Motivation

The MAF/TIGER (Master Address File/Topologically Integrated Geographic Encoding and Referencing) system has supported the Census Bureau’s data collection, tabulation, and dissemination since the 1990 decennial census. Created in the 1980s through a partnership between the Census Bureau and the United States Geological Survey (USGS), MAF/TIGER has since been “the sole source for all maps, address information, and geographic reference data” underlying the Bureau’s operations^8^.

Initial use cases for the MAF/TIGER required exact topological accuracy but little positional accuracy. For topological accuracy addresses needed to be assigned the correct geographic codes (e.g., census block, tract, county). But less strict requirements for positional accuracy meant that the geographic structure and shape of units for which the Bureau published data needed to be recorded, but only with respect to other represented features, not exact locations on the Earth^9^. As long as survey responses were tabulated in the correct geographic unit and all survey responses were uniquely assigned to a unit, the system functioned as required.

The 1990 census was supported by the initial MAF/TIGER database, and the 2000 decennial census was supported by a MAF/TIGER database that had been incrementally improved during the 1990s. During that decade, the Bureau added new addresses and geographic features to support its operations, but these additions still required only topological accuracy. For most features, the positional accuracy remained about the same.

Throughout the 1990s, users inside and outside the Census Bureau began to realize the potential of the MAF/TIGER database for broad-ranging applications beyond its initial purpose, but many of these applications required a higher level of positional accuracy. External users began using TIGER/Line files—the public files derived from MAF/TIGER—for large-scale mapping and geocoding. Internally, the Bureau wanted to support its operations by capturing the latitude and longitude of every dwelling unit in the United States using GPS, and they wanted to integrate local and tribal GIS data into its processes^9^. To achieve the level of positional accuracy that these applications demanded, the Bureau planned and executed the MAF/TIGER Accuracy Improvement Project (MTAIP)^8^.

Completed between 2002 and 2008, the MTAIP’s goal was the creation of a seamless, national database with road centerlines required to be positionally accurate within 7.6 meters (25 feet) at most. Integrating GIS data from governmental entities, commercial firms, aerial imagery, and GPS traces, the MTAIP improved the positional accuracy for data in all 3,141 counties and county equivalents in the U.S., and it instituted practices for data collection that continued in subsequent years^8^. The Bureau then used the more accurate MAF/TIGER data for the 2010 decennial census.

**Fig. 1:**
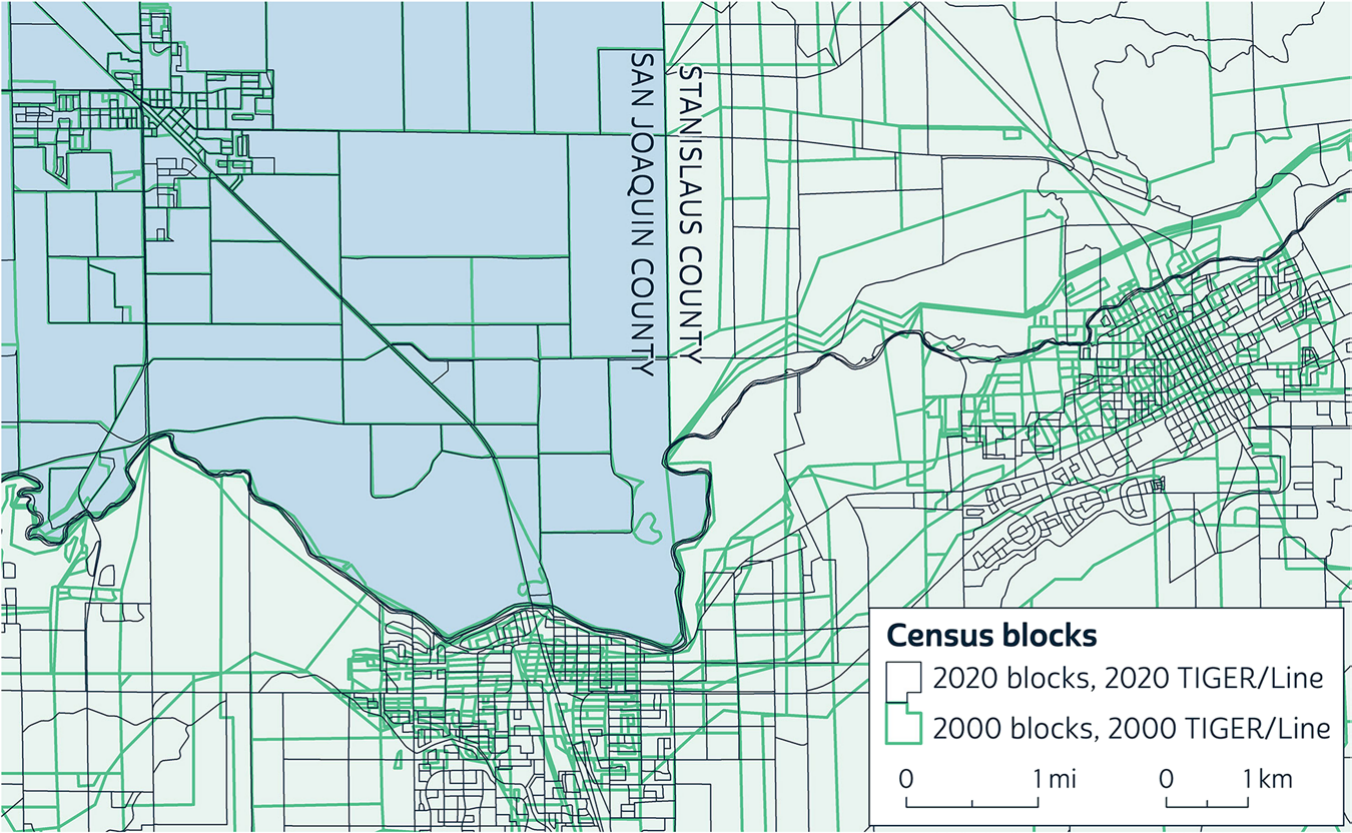
Extreme discrepancies in census block representations in California. Source: NHGIS.

Stanislaus County, California, offers an extreme example of the MTAIP’s effects (Fig. 1). Between 2000 and 2020, most of the county’s block boundaries remained unchanged *on the ground*, aside from a few growing areas where new blocks were added. But there were many large changes in the *representations* of block boundaries, correcting for poor positional accuracy in 2000. The only boundary lines in Stanislaus County that the MTAIP did not shift significantly are along the boundary with San Joaquin County, where the 2000 representations were relatively accurate. For example, where the Stanislaus River runs along the county boundary to the west, the distances between the 2000 and 2020 representations are generally small. In contrast, farther east, the river’s representation within Stanislaus County shifted as much as 750 meters to the south in some places, along with the entire city of Oakdale. The shifts in San Joaquin County are far less dramatic, but they are still pervasive, affecting nearly every block there as well. The comparison shows that the variation in the quality of the Census Bureau’s spatial data was both significant and uneven in space, creating many opportunities for significant error in comparisons across time.

While the MTAIP made substantial improvements to the TIGER database, it made relatively few improvements directly to the MAF, the official register of addresses and geographic coordinates of housing units and group quarters^10^. Subsequent Bureau programs have therefore aimed to improve the quality of the MAF, beginning with work carried out during address canvassing for the 2010 census. Over 140,000 people^11^ walked or drove all streets in the United States comparing addresses against the MAF and, crucially, capturing GPS coordinates for each address, which were then recorded in the MAF^12^. Afterward, one of the core initiatives of the Bureau’s Geographic Support System (GSS) in the 2010s was MAF improvements, including a process to continually update the MAF outside of decennial census operations^10^.

While the ongoing improvements have ensured that new census data products now offer consistently high reliability, the earlier products remain unaltered, so researchers undertaking retrospective or longitudinal analysis must contend with both the widespread inaccuracies in older products and their inconsistency with newer products. Numerous data sets provide spatially standardized data for longitudinal population research, including contributions from the IPUMS National Historical Geographic Information System (NHGIS)^13^, two contributions from Professor John Logan (Brown University) with various collaborators^6,14^, and the Neighborhood Change Database from GeoLytics. Recent additions oriented toward housing units^15^ and economic opportunity^16^ offer their own advantages in terms of focus and precision. Leyk *et al*. review over a dozen gridded population estimate products, and their list is still only a partial look at the various products assembled to allow for global analysis of populations^17^. This is an impressive list of data products supporting an even more extensive range of research. Virtually all of them rely, to some degree, on population counts produced with census data for which the positional accuracy of older data has not been revised. However good the efforts are to produce accurate counts from these data, they cannot eliminate deficiencies due to the underlying positional errors.

The inconsistent levels of positional accuracy associated with even relatively recent Census products represents a significant issue in the U.S. federal data infrastructure and a barrier to accurate longitudinal analysis for phenomena that vary at fine spatial scales. From measures of segregation^18^ to the health effects of air pollution^19–21^ very small differences in how populations are allocated can impact results. Importantly, the effects of positional accuracy vary significantly across urban and rural places and across decades meaning that the error introduces structured bias with respect to spatial and longitudinal processes. Fixing this source of bias and error represents a significant step forward for Census data infrastructure for researchers conducting analyses that rely on precise geographies.

## Methods

This project was conducted entirely within the secure research environment of the FSRDC system; thus, we leave some of the details of the restricted-access data intentionally vague to comply with the privacy requirements imposed by federal law.

### Data

The Census Bureau provided us with all person and household responses collected during the 1990, 2000, and 2010 decennial censuses. The 2000 and 2010 records contain a Master Address File ID (MAFID) that associates each response with a record in the MAF. The MAF in turn identifies the street address and geographic coordinates (longitude and latitude) for each housing unit and group quarters included in the census.

The 1990 census records include no MAFID and therefore required additional effort to link to later spatial information. The Bureau produced a data set like the MAF in 1990 called the Address Control File (ACF), which includes street addresses for nearly all 1990 census responses but lacks geographic coordinates. To link 1990 responses to the spatial information available in the MAF, we used an ACF-MAF crosswalk that Katie Genadek, J. Trent Alexander and collaborators developed by matching address strings in the two products. This crosswalk resides within the FSRDC environment and was a component of their broader efforts to link individual respondents across censuses from 1940 to 2020^22,23^. The crosswalk is not complete, but it provides matches to the MAF for roughly 70% of 1990 respondents, using not only address descriptors to make linkages but also other respondent information and administrative data (such as tax returns) that were not available to our research team.

The MAF is a dynamic database, continually evolving with new updates. As such, the Bureau does not provide researchers direct access to it but instead produces annual snapshots, called Master Address File Extracts (MAFX). Each MAFX contains the addresses and coordinates that were in use at the time of the extract. The Bureau provided us with access to the MAFX for most years between 2008 and 2020 (2008–2012, 2014, 2017, 2019, and 2020).

A second key piece of spatial information included in both the MAFX files and the census responses is the tabulation block ID, which identifies the census block where each address/response was tabulated in data products for the corresponding year. For example, the tabulation block IDs in the 2000 MAFX and census responses identify the 2000 census blocks where responses were tabulated for 2000 census data products.

We used block-level GIS-compatible boundary information for 1990, 2000, 2010, and 2020 from NHGIS^13^. The NHGIS boundaries are derived from TIGER/Line files and therefore generally correspond properly with the spatial information provided in MAFX files. We used water area features from the 2000 and 2010 TIGER/Line files to distinguish land areas within blocks, which allowed us to avoid placing coordinates in water features.

### Process

**Fig. 2:**
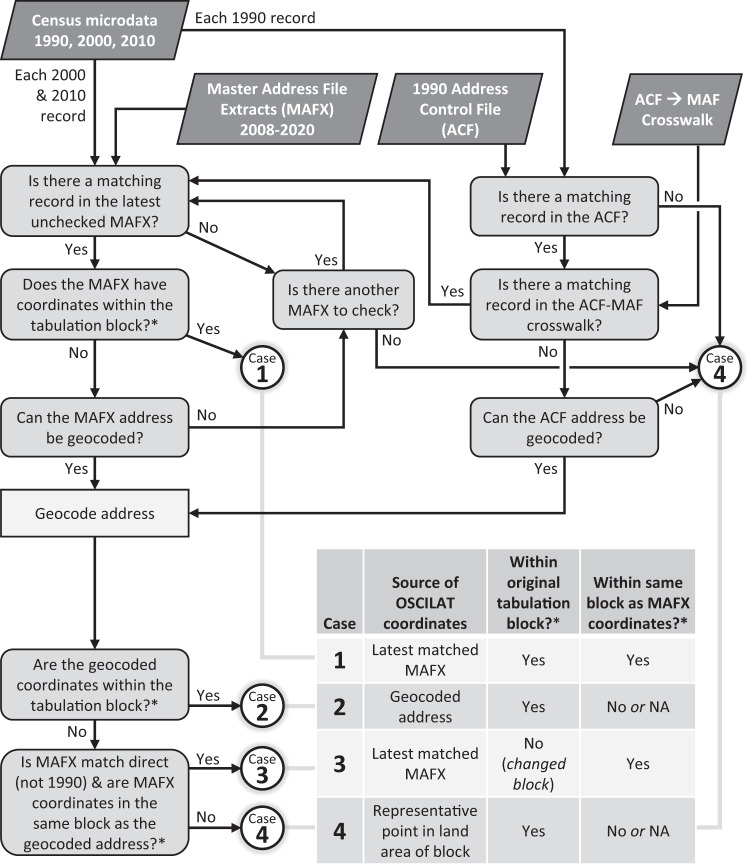
Generalized workflow for assigning OSCILAT geographic coordinates. *To determine whether coordinates are “within a block”, we limited the comparisons to land area (excluding water), and we applied special handling for 1990 blocks due to the poor quality of available 1990 block boundary information (see text for details).

**Fig. 3:**
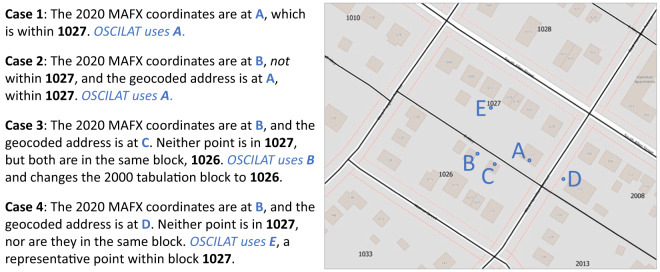
Hypothetical illustration of the four OSCILAT assignment cases for a 2000 census response with a tabulation block ID of 1027 and a MAFID that matches with a record in the 2020 MAFX.

To optimize the latitude and longitude for each census response, our principal strategy was to use coordinates from the most recent MAFX file that we could match to the microdata record. We assume the most recent MAFX file to be more accurate than earlier MAFX files due to the Bureau’s continual improvement of the MAF over time. Crucially, we also aimed to achieve agreement between each record’s coordinates, tabulation block, and street address. Due to limitations of our sources, we were sometimes unable to match a microdata record to a MAFX file, and sometimes the corresponding MAFX coordinates were not consistent with the corresponding tabulation block boundaries or the available address information. Our process distinguished four distinct cases of information agreement and assigned optimized spatial information differently for each (Figs. 2, 3).

In brief, the key steps in the coordinate assignment process were:

1. Attempt to link a record in the microdata to a record in the MAFX starting with the most recent MAFX (2020) and working backwards until we find a match.
2. If the matched MAFX coordinates fall within the tabulation block, use them (*Case 1*).
3. Otherwise, geocode the available address and:
  - If the geocoded coordinates fall within the tabulation block, use them (*Case 2*).
  - Otherwise, if the geocoded coordinates fall within the same block as the MAFX coordinates, use the MAFX coordinates, in effect changing the block associated with the census response (*Case 3*).
4. If all of the above fails, use coordinates for a representative point within the tabulation block (*Case 4*).

In the first, preferred case (Case 1), we were able to match the microdata record to a MAFX record (by MAFID or, in the case of 1990 data, through the ACF-MAF crosswalk), and the coordinates from the latest matching MAFX were located within the tabulation block. To determine whether coordinates were located “within a tabulation block,” we applied a spatial join between the coordinates and water-clipped block boundaries, ensuring that the coordinates were located within the land area of the block. In all of these cases, the OSCILAT coordinates come from the latest matching MAFX, and there is no change in the tabulation block. This was by far the most common case, including about 96% of 2010 records, 90% of 2000 records, and 70% of 1990 records.

If the Case 1 conditions were not met, but address information was available (e.g., where the matching MAFX records were missing coordinates or the given coordinates fell outside the tabulation block), we would then geocode the address information. We used the geocoding engine associated with the SAS statistical software with street geometry and address range information from the 2010 TIGER/Line files and address information from the most recent matching MAFX or, in the case of 1990 records, from the Address Control File (ACF). Where SAS was able to geocode the address (i.e., it was able to associate the address with a specific location along a street and return geographic coordinates), we tested the coordinates against the water-clipped tabulation block boundary. From here, we looked for points of agreement. If the geocoded point fell within the tabulation block’s land area, we retained the geocoded coordinates and the originally assigned block ID (*Case 2*). For 2000 and 2010 U.S. records, if the geocoded coordinates fell outside the tabulation block’s land area but lay in the same block as the MAFX coordinates, we retained the MAFX coordinates and assigned a new block ID (*Case 3*). (For 1990 records, we skipped Case 3 assignments because we rated the reliability of the original tabulation block ID higher than the reliability of the address string matching used to link 1990 addresses to MAFIDs in the ACF-MAF crosswalk. Similarly, we skipped Case 3 for Puerto Rico records due to the uncertain reliability of the latest MAFX files there).

If none of the conditions were met for any of the first three preferred cases (e.g., neither the geocoded nor MAFX coordinates fell within the tabulation block or in the same block as each other, or if there was no matching record with valid coordinates or a geocodable address in either the MAFX files or the 1990 ACF), we then assigned coordinates based on a ‘representative point’ within the land area of the tabulation block (*Case 4*). To identify a block’s representative point, we applied the GeoPandas representative_point() function to the water-clipped block polygon. This function places the point at the block centroid unless the centroid falls outside the block’s land area, in which case it uses the midpoint of the longest internal segment of a line drawn across the polygon. This case was uncommon for 2010 (1.2% of records) and 2000 (2.8%) but unfortunately somewhat common for 1990 (24%). These are all cases where the internal census data sources provide no reliable spatial information more precise than a block ID. Using a land-based representative point at least ensures that the census response is located on land at or near the mid-point of the block where the response was tabulated.

Our process also involved special handling to address the poor quality of available information on 1990 block boundaries. The 2010 TIGER/Line files, which include the major accuracy improvements achieved by the MTAIP, include boundary lines for 2010 and 2000 census blocks, but the best available boundary information for 1990 blocks comes from the poorer-quality 2000 TIGER/Line files. If we used boundaries from the 2000 TIGER/Line files to validate coordinates, it would frequently occur that accurately placed points would lie *outside* of the correct corresponding block due to the boundaries’ poor positional accuracy. Therefore, we used 2000 TIGER/Line files only to determine *topological relationships* between 1990 and 2000 blocks. Then to check whether coordinates for a 1990 record were in the correct corresponding 1990 tabulation block, we did not spatial join directly to boundaries for that 1990 block. Instead, we checked against 2010 TIGER/Line boundaries for the set of *2000 blocks* that share land area with the 1990 block (according to 2000 TIGER/Line topological relationships). In addition, to produce representative points for 1990 blocks (for Case 4 assignments), we applied a multi-stage subprocess using both 2000 and 2010 TIGER/Line boundaries to shift 1990 block centers as needed to ensure that each final 1990 representative point is located within 2010 TIGER/Line definitions of the land areas of 2000 blocks that share land area with the 1990 block.

Finally, once we assigned optimized coordinates, we conducted a spatial join to assign each census response the corresponding 2010 and 2020 block IDs to facilitate longitudinal analysis.

## Results

The OSCILAT project assigned consistent latitude, longitude, and block IDs to nearly 1.2 billion Census microdata records covering all population and housing responses to the 1990, 2000, and 2010 decennial censuses (Table 1 “Total Records”). Table 1 offers a summary of the methods used to make these assignments, which relied on data from multiple MAF extracts, the use of modern geocoding techniques, and improved representations of boundaries.

**Table 1:** Counts and rates of OSCILAT assignment cases, rounded for disclosure avoidance.

Year	Data Type	Total Records	Case 1. Latest MAFX, Same Block		Case 2. Geocoded, Same Block		Case 3. Latest MAFX, Changed Block		Case 4. Representative Point in Block	
			N	%	N	%	N	%	N	%
2010	Population	312,473,000	300,809,000	96.3	6,253,900	2.0	1,716,950	0.5	3,701,300	1.2
	Housing	133,512,000	128,180,000	96.0	2,949,600	2.2	767,150	0.6	1,610,600	1.2
2000	Population	285,222,000	256,386,000	89.9	13,303,000	4.7	7,572,400	2.7	7,968,000	2.8
	Housing	117,320,000	105,320,000	89.8	5,837,350	5.0	3,058,100	2.6	3,108,200	2.6
1990	Population	248,714,000	174,576,000	70.2	15,066,000	6.1	*	*	59,070,000	23.8
	Housing	102,401,000	70,279,500	68.6	6,767,400	6.6	*	*	25,350,900	24.8

*OSCILAT does not alter original block IDs for 1990 records due to uncertainty in linkage between 1990 records and MAFX.

## Discussion

Users of OSCILAT should be aware that even these “optimized” coordinates may include instances of substantial positional inaccuracy, particularly for the Case 4 instances based on representative points within blocks. Still, importantly, all four of the main OSCILAT assignment cases represent an improvement over the legacy information. The only setting where the improvements may be considered generally minor is for Case 1 in 2010, for which the information from the latest MAFX was sometimes only a confirmation of what was employed at the time of enumeration, with potentially little or no improvement in precision. For 2000, Case 1 improvements were generally more significant, as most 2000 survey responses had valid MAFIDs but were assigned locations with comparatively imprecise locations. By definition, these Case 1 records did not shift to a new block from their 2000 assignment, but their improved precision can be significant in determining how they are assigned to other geographies such as those for 2020 blocks. The largest source of improvement, however, comes in the 1990 data where the many Case 1 records gained a level of precision that simply did not exist for 1990 responses. These responses only ever had a block ID and possibly a valid street address but lacked coordinates altogether. Matching them to the latest MAFX represents a significant enhancement. Once again, we must credit Genadek and Alexander for matching 1990 records to MAFIDs, which drove this portion of the process for 1990^22,23^.

Case 2 assignments involved the addition of coordinates based on a geocoded address to census responses where no valid coordinates previously existed. These records either had coordinates that fell outside the tabulation block’s land area or had no coordinates provided at all. OSCILAT records in this category now have detailed spatial information that puts them on par with records already supported with correct information in the MAFX.

**Fig. 4:**
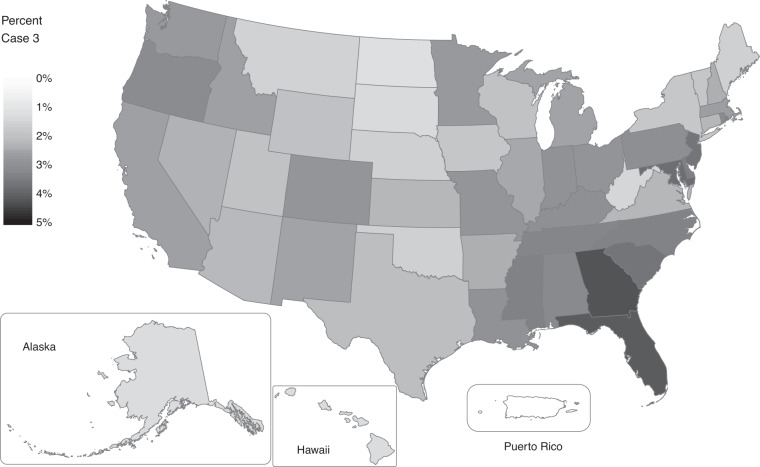
Share of 2000 person records with a changed block ID in OSCILAT (Case 3).

Case 3 encompasses a set of 2000 and 2010 records where we had sufficient information from the MAFX and from geocoding to justify changing the block ID of a census response. While these represent a relatively small portion of the overall records, they are significant because they will result in changes in population or housing counts within small geographies. For the 2000 census, we changed the block ID for about one of every 38 records (2.6%), representing a sizeable error in the original 2000 tabulations. For these cases, subsequent improvements in the MAF/TIGER system indicate that the 2000 responses should, in fact, be counted in different blocks than those in which they were originally counted. Moreover, the distribution of this error was not random in space but concentrated in certain kinds of places as shown in Fig. 4. Rates were highest in Georgia and Florida where they exceeded 4%. (There is no variation in Puerto Rico where, as with 1990 records, there was not sufficient information to support block ID changes.) The rates of variation at the county and tract level are higher still and reflect significant variations in quality between rural and urban places that could substantially influence outcomes for analyses conducted at finer geographic scales. The case assignment codes in the OSCILAT data allow users to identify exactly where these cases occur and investigate possible implications for their own analyses.

**Fig. 5:**
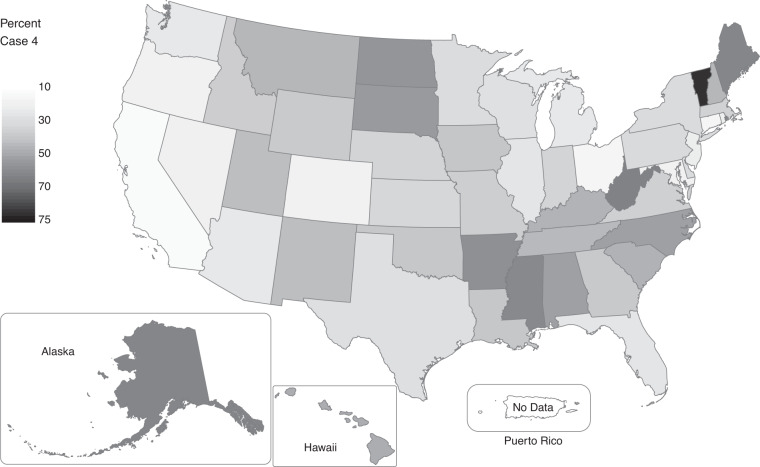
Share of 1990 person records with OSCILAT locations based on block representative points (Case 4).

Case 4 assignments have the weakest precision of the four cases. Using a land-based representative point within a block ensures only that the OSCILAT coordinates are within the same block where the census response was tabulated, which should be effective for studies of larger areas but would be problematic for studies that require differentiation of populations *within* blocks, e.g., to identify households residing in a flood zone or on high-traffic roads. Case 4’s liabilities will also be more severe in rural areas because rural blocks may be very large, causing larger potential displacements between representative points and actual residences. This is particularly of concern for 1990 data because match rates between 1990 records and MAFX files were considerably lower in rural areas, resulting in high rates of Case 4 assignments there. Figure 5 demonstrates how 1990 state-level Case 4 assignment rates are highly correlated with the rurality of the states. Rates range from 5.9% in the entirely urban District of Columbia to 68% in very rural Vermont, with several other heavily rural states also having rates over 50% (in descending order: West Virginia, Alaska, Maine, Mississippi, Arkansas, and North Dakota). These high rates are generally due to rural areas having less reliable and/or outdated street address information in 1990, making it more difficult to find matches either in the MAF or through geocoding.

Whatever the liabilities of Case 4 assignments, they still represent a significant improvement over previously available spatial information. As noted above, these are all cases where the internal census data sources provided no reliable spatial information more precise than a block ID; using a land-based representative point at least ensures that the census response is placed on land at or near the mid-point of the block where the response was tabulated. This allows for a complete tabulation of earlier censuses within arbitrary boundaries and ensures that census responses with limited location information are still included in spatial analyses employing OSCILAT. Case 4 liabilities are also likely to have minimal effects on analyses within urban areas because Case 4 assignments are relatively uncommon in urban areas in 1990, and the generally smaller sizes of urban blocks ensures that their representative points will be nearer to residences in absolute terms. As with Case 3, the OSCILAT case assignment codes will allow users to identify where Case 4 assignments occur and investigate possible implications for those areas.

## Data Availability

To summarize OSCILAT data access protocols, the data is available in the data warehouse within the FSRDC system for use in approved projects vetted through a standardized application process^24^. Within the census FSRDC we provide separate files for population and housing records for each combination of census (1990, 2000, and 2010) and state (plus the District of Columbia and Puerto Rico but excluding outlying territories). Each file contains the unique linking record ID used in the census microdata, the optimized latitude and longitude, the block ID for that survey year (the original tabulation block for 1990 or the optimized block based on optimized coordinates for 2000 and 2010), and optimized 2010 and 2020 census block IDs for all survey years. Records also include the coordinate assignment case number approximately corresponding to the cases identified here in Fig. 2 and Table 1. In practice, we use a larger number of codes than the four presented here to account for a range of edge cases and some particulars for specific years. These are documented in the data dictionary that accompanies the files in the FSRDC data warehouse but omitted here because of the need to simplify the number of cases for disclosure purposes. Users of the data must request access to both the appropriate census microdata and the OSCILAT data. OSCILAT only includes linkages to the censuses, not the responses themselves. Conversely, users who only need records for a specific state, year, or class of response (population or housing) could request the specific state/year/class they need and further reduce their data request, increasing the likelihood that it will be approved by Census.

## Code availability

Code used to generate the optimized coordinates for OSCILAT is not retained or distributed with the data in the data warehouse because of the way that Census firewalls its restricted data. The approval process for projects within the FSRDC is designed to minimize researchers’ access to only the information needed for their specific project. Because it retains only the bare minimum of information necessary to link census responses to standardized location information, access to OSCILAT is substantially easier to obtain than access to all of the MAFX and census responses used to generate OSCILAT. Because the code to generate OSCILAT responses includes information about those data sets it would only be accessible to individuals who had gained approval for all of the census responses, MAFXs, and the ACF. Rather than impose this burden on OSCILAT users, the code is retained only within the FSRDC project space.
